# Multifaceted Regulation of Tea Astringency: A Review of Intervention Pathways by Co-Existing Flavor Components

**DOI:** 10.3390/molecules31203659

**Published:** 2026-10-13

**Authors:** Shikun Liu, Tianhui Huang, Yifei Zhang, Jiale Kuang, Xianghao Wu, Weijia Li, Hongwei Yu, Jingjing Liu

**Affiliations:** College of Automation Engineering, Northeast Electric Power University, Jilin City 132012, China; bearyeah2022@163.com (S.L.); 13756468775@163.com (T.H.); 1202500141@neepu.edu.cn (Y.Z.); 2202300748@neepu.edu.cn (J.K.); 2202500820@neepu.edu.cn (X.W.); liwj@neepu.edu.cn (W.L.); yuhw@neepu.edu.cn (H.Y.)

**Keywords:** astringency, polyphenol-protein interaction, tea, flavor components

## Abstract

Tea, as a widely consumed natural beverage, has long been a focus of sensory quality research due to its harmonious and layered flavor. Among the complex flavor characteristics of tea, astringency is one of the key attributes determining the overall tea flavor, and its generation is closely related to the interaction between polyphenolic compounds and salivary proteins. Phenolic hydroxyl groups and benzene rings in the molecular structure of polyphenols can bind to salivary proteins via non-covalent interactions to form insoluble complexes, which can alter oral lubrication and elicit astringency. However, the tea astringency is not only determined by polyphenols in tea but also modulated by flavonol glycosides, alkaloids, amino acids, polysaccharides, and other flavor components. Depending on differences in functional group composition and spatial structure, these flavor components act on different stages in the formation of astringency, and ultimately affect the astringency expression. These regulatory pathways can be categorized into three types based on their stages and targets: protein-side regulation, polyphenol-side regulation, and complex aggregation process regulation. This review systematically summarizes the molecular mechanisms, representative components, and regulatory effects of these three categories, aiming to provide theoretical support for the targeted modulation of tea flavor quality and offer insights for research on other astringent foods.

## 1. Introduction

Tea occupies an important position in food flavor research due to its diverse chemical composition and complex sensory characteristics. Among the many flavor attributes of tea, astringency is one of the key factors influencing tea infusion quality evaluation [1]. The astringency in tea originates mainly from catechins and their derivatives, which contain phenolic hydroxyl groups and benzene rings. The loss of oral lubrication resulting from their binding to salivary proteins is the key mechanism for generating astringent sensations such as dryness and puckering [2,3]. However, in addition to tea polyphenols and other astringent components, there are flavonol glycosides, alkaloids, amino acids, polysaccharides, and other chemical components that exhibit other taste attributes [4,5,6]. The formation of astringency in tea relies on weak, reversible, non-covalent bonds between polyphenols and salivary proteins, making the process susceptible to interference from other coexisting components. Therefore, the final astringent intensity and sensory characteristics exhibited by tea are not determined by tea polyphenols alone but result from the combined regulation of these coexisting flavor components.

At present, most reviews on tea astringency focus on changes in flavor component contents, processing procedures, and the relationship of these factors to final astringency characteristics. They mainly discuss how different chemical components affect the astringency of tea through interconversion or changes in content [7,8,9]. There is still a lack of systematic summary of how other flavor components in tea regulate astringency through polyphenol-protein interactions. Accordingly, this review focuses on the main targets and action stages of tea flavor components involved in polyphenol-protein interactions and summarizes the astringency regulation mechanisms.

The flavor components in tea vary significantly in functional group composition, molecular size and spatial structure. Some of them possess aromatic rings and phenolic hydroxyl groups; some contain polar groups such as carbonyl, amino and carboxyl groups; and some have macromolecular chain segments and charge characteristics [10]. It is these structural differences that enable them to interact with polyphenols, salivary proteins, or complexes of both in different ways, thus allowing them to participate in the modulation of tea astringency intensity through distinct mechanisms. In terms of the stage of action, some components primarily act at the polyphenol-protein binding stage, thereby regulating complex formation by affecting the availability of salivary proteins or the binding state of polyphenols. Others primarily act on processes after complex formation and aggregation, influencing astringency expression by altering complex stability, aggregation state, or oral lubrication.

Therefore, this review systematically searched the Web of Science and Google Scholar databases using search terms related to astringency perception, such as “tea astringency” and “astringency perception”, and to the modulation of astringency by tea flavor components, for example: “flavor components in tea”, “flavonol glycosides and protein”, “alkaloid and polyphenol”, “amino acid and polyphenol”, and “polysaccharide in tea”. Studies that could provide mechanistic evidence for the modulation of astringency by flavor components were selected, while letters and conference papers were excluded. Finally, through reviewing and analyzing these studies, this review investigated the molecular interactions among several common flavor components in tea and the role of polyphenol–protein binding in astringency perception mechanisms. From a mechanistic perspective, the regulation of astringency perception by common tea flavor components was categorized into three pathways: protein-side regulation, polyphenol-side regulation, and regulation of the complex aggregation process. This review systematically introduces the regulatory effects of these flavor components on astringency within this mechanistic framework, aiming to provide a theoretical reference for analyzing the complex causes of astringency in tea. This exploration of regulatory mechanisms is not only applicable to the tea matrix but also serves as a reference for the study of other astringent foods with complex flavor compositions, providing a valuable approach for regulating the astringency properties of foods.

## 2. Formation of Astringency in Tea

Catechins and their derivatives are major sources of astringency in tea. These polyphenols bind to salivary proteins and induce astringency by increasing oral friction. However, in tea, a complex system rich in multiple flavor components, the intensity and sensory characteristics of astringency are not determined solely by astringent components but are also affected and modulated by other flavor components. This modulation may be associated with the functional group composition, spatial structure, and molecular size of flavor components, as well as their influence on the state of complexes, thereby altering the process of astringency formation at the molecular or colloidal level. Therefore, before systematically exploring how other flavor components regulate tea astringency through multiple pathways, it is necessary to clarify the material basis and perception mechanisms of astringency itself. On this basis, this chapter mainly summarizes the structural characteristics of common astringent compounds in tea and further discusses the mechanisms related to astringency perception, such as polyphenol–salivary protein interactions, loss of oral lubrication, and structural changes in these compounds.

### 2.1. Relevant Components

**Figure 1:**
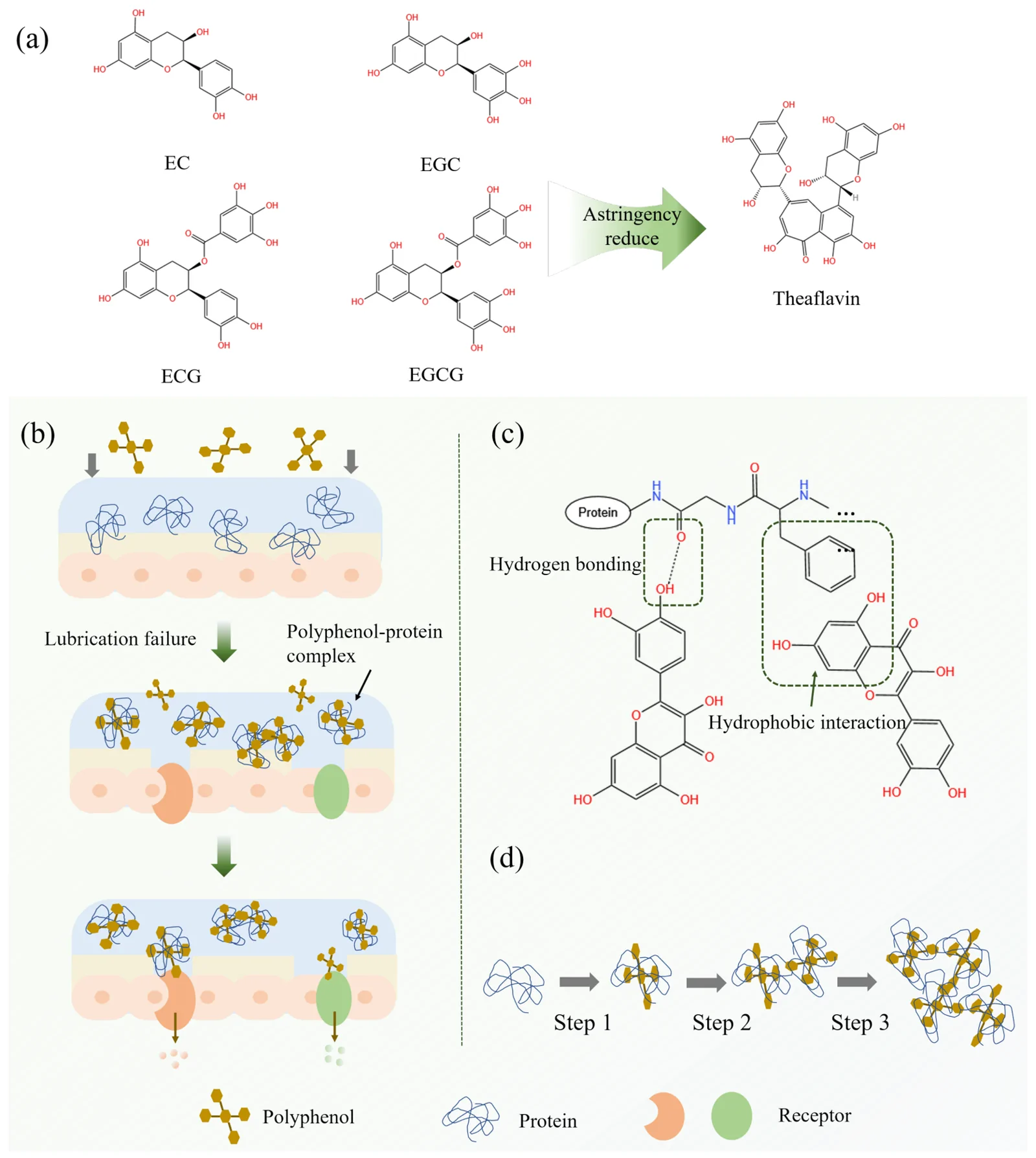
(**a**) Chemical structures of common tea polyphenols; (**b**) Schematic of lubrication failure mechanism; (**c**) Schematic of non-covalent binding between protein and polyphenols; (**d**) Three-stage model of protein-polyphenol binding.

Early tannase hydrolysis experiments suggested that gallic acid exists in tea as ester bonds, indicating that some tea polyphenols contain gallic acid ester structural units [11,12]. Subsequent crystallization analysis of tea further revealed that tea also contains catechin, epicatechin, and other tea polyphenols without gallate structures [13]. To date, more than 30 types of tea polyphenols have been identified, with (-)-epicatechin (EC), (-)-epigallocatechin (EGC), (-)-epicatechin gallate (ECG), and (-)-epigallocatechin gallate (EGCG) being the most common [14]. The structures of these compounds are shown in Figure 1a.

From the structural type, tea polyphenols can be classified into catechins and their derivatives. Structurally, these catechins share certain common features, that is, they all contain two benzene rings (A ring and B ring) and a dihydropyran heterocyclic ring (C ring), and the C3 position is substituted by hydroxyl [15,16]. These structural units are closely associated with the protein-binding ability of catechins. The hydroxyl group acts as a hydrogen bond donor for catechin-protein binding, and the benzene ring provides a site for hydrophobic interactions. Therefore, catechins generally possess protein-binding ability and thus constitute an important material basis for the formation of tea astringency.

However, catechins differ structurally, mainly in the number of hydroxyl groups on the B ring and in whether the C3 hydroxyl forms an ester bond with gallic acid. The more hydroxyl groups there are, the more sites are available for hydrogen bonding [17]. Galloylated catechins have three additional hydroxyl groups compared with their non-galloylated counterparts [18]. An increase in the number of hydroxyl groups means that the molecule has more potential sites for hydrogen bonding, which may enhance its ability to bind to proteins. Thus, galloylated catechins, or catechins with more hydroxyl groups, generally exhibit stronger interactions with proteins [19,20]. The relationship between structure and activity mentioned above suggests that the astringency of tea is related not only to the content of tea polyphenols but also closely to the specific structures of tea polyphenol molecules and their protein binding ability. Differences in catechin composition and content among tea varieties may further lead to differences in tea infusion astringency.

### 2.2. Formation Mechanism

As mentioned above, astringency can be defined as an oral sensation caused by the mechanical deformation of the oral epithelium induced by astringent compounds such as tannins [21]. This definition indicates that although astringency is usually classified as a taste or oral sensation, its perception is related to the state of the oral epithelium, changes in lubrication, and mechanical friction [22]. In the tea matrix, the loss of lubrication caused by the binding of astringent components such as catechins to salivary proteins is one of the key mechanisms underlying the perception of tea astringency [23]. Owing to the complex structure of the oral salivary film, the loss of lubrication is not a single, instantaneous process but may occur in phases [24,25,26,27]. The schematic diagram is shown in Figure 1b. The salivary film consists of two layers: the fluid saliva layer, which is mainly composed of proline-rich proteins (PRPs) involved in polyphenol binding, and the mucosal layer cross-linked to the oral epithelial cells, which is mainly composed of mucins involved in polyphenol binding. When polyphenols enter the oral cavity, they first bind to PRPs in the flowing saliva layer, forming complexes that progressively aggregate into insoluble complexes. This process consumes PRPs in saliva, decreases the thickness of the hydration layer, increases interfacial roughness, and sharply decreases the film thickness ratio, indicating that the lubrication state in the oral cavity changes from mixed lubrication to boundary lubrication. Then, the remaining polyphenol molecules continue to diffuse into the mucosal layer, bind to mucins therein, and destroy the complete structure of the salivary membrane. This process indicates that the effective lubricating film is destroyed and its protective function is lost in the oral cavity, causing boundary lubrication failure. This process shows that polyphenol-protein interactions are the core link in the lubrication failure mechanism, and it is also an important basis for understanding how other flavor compounds modulate astringency formation [28,29].

Carbonyl groups and nonpolar side chains in salivary proteins can bind to phenolic hydroxyl groups and benzene rings in the chemical structure of polyphenols through hydrogen bonds and hydrophobic interactions, respectively, to produce polyphenol-protein complexes [30,31]. The binding process is illustrated in Figure 1c. It is worth noting that these complexes are not completely insoluble, and its solubility depends on the ratio of polyphenols to proteins. Research on the self-diffusion coefficient during the binding process between proteins and polyphenols reveals that changes in the solubility of polyphenol–protein complexes follow a three-stage model, as shown in Figure 1d. When the binding sites of polyphenols are far fewer than those of proteins, they first form polyphenol–protein monomer complexes. As the amount of polyphenols increases, the number of available binding sites increases, and polyphenol–protein complexes begin to associate to form dimers until a certain equilibrium is reached. As polyphenol concentration continues to increase, these dimers further associate, eventually forming larger polymers and precipitating [32].

This three-stage model lays the foundation for understanding differences in perceived astringency intensity. If only the effects of astringent components are considered, perceived astringency intensity is closely related to the polyphenol–protein ratio. Therefore, in addition to differences in polyphenol concentration, salivary flow rate also plays a role [33], and basal salivary protein concentration [34] can affect the perceived intensity of astringency. Generally speaking, individuals with a high salivary flow rate are better able to dilute and flush out astringent components entering the oral cavity, resulting in lower perceived astringency intensity at a given concentration. Moreover, interindividual differences in basal salivary protein concentration can affect the sensitivity to astringency perception.

In addition, the binding of polyphenols to proteins primarily relies on non-covalent interactions, this also implies that other flavor compounds in tea with similar structural units or binding properties may intervene in the binding process of polyphenols to proteins through mechanisms such as competition or interference, thereby regulating the final astringency expression.

## 3. Other Components Regulating Tea Astringency

Although catechins are key to the mechanism underlying tea astringency, tea astringency is not solely determined by compounds such as tea polyphenols but rather the result of the combined effects of various flavor components [35]. In the tea quality evaluation standard based on sensory description (GB/T-23776), tea infusion taste can be described using terms such as “refreshing“, “mellow“, “sweet and fresh“, “greenish and astringent“, and “strong and astringent“ [36]. These descriptions indicate that differences in both intensity and texture, and this variation is largely closely related to the involvement of other flavor components in tea infusion. Currently, hundreds of flavor compounds have been detected in tea. Besides tea polyphenols, many other components can contribute to sensory characteristics such as sweetness, bitterness, and umami, or even attributes such as smoothness, richness, and refreshing character. These components coexist in the tea infusion, and due to their chemical structure, they may interact with one another. To more clearly demonstrate the regulatory effects of common flavor components on astringency, this review focuses on flavonol glycosides, alkaloids, amino acids, and polysaccharides, which are among the most representative and abundant flavor components in tea [37,38,39]. It considers only the regulatory effects of individual components on astringency and explores how these components participate in astringency formation from a molecular mechanism perspective. Table 1 presents several common flavor components in tea and their taste characteristics. These flavor components collectively shape the overall flavor of tea by suppressing or enhancing astringency.

Flavonol glycosides are among the important flavor-related components in tea. As a common subclass of flavonoids, flavonol glycosides are widely present in nature [40,41]. Flavonol glycosides are typically composed of flavonol aglycones linked to various types or numbers of glycosyl groups, which can give rise to 200~300 distinct combinations depending on the length or sequence of the glycosyl chains. Common aglycones include quercetin and kaempferol, whose structural formulas are shown in [42]. The phenolic hydroxyl groups can act as hydrogen bond donors, allowing flavonol glycosides to bind to the limited number of binding sites on salivary proteins. This enables them to elicit astringency through lubrication failure. However, many sensory studies on flavonol glycosides suggest that they exhibit a delicate, silky astringent taste characteristic [43,44]. This is because flavonol glycosides typically lack galloyl groups, and the reduction in the number of hydroxyl groups results in milder astringency, thus producing a gentler, silkier texture. Moreover, the chemical structure of flavonol glycosides gives them the potential to modulate polyphenol–protein interactions. Not only can they impart a milder astringency to tea infusion, but they may also alter the texture and persistence of the astringency in the tea infusion through interactions with salivary proteins.

Alkaloids are also abundant in tea, with common examples including caffeine, theobromine, and theophylline. They share a xanthine skeleton and can be distinguished by the number of methyl groups and their substitution positions [45,46]. These alkaloids can activate receptors such as TAS2Rs to elicit bitterness, characterized by fast onset and slow decay, which imparts a lingering bitterness to tea infusion. However, their flavor contribution in tea is not limited to bitterness [47,48,49]. For example, caffeine, the most abundant and extensively studied alkaloid in tea, has the structure 1,3,7-trimethylxanthine, and its purine ring enables the possibility for π-π stacking interactions with polyphenols [50]. Therefore, alkaloids such as caffeine act on the polyphenol side and indirectly affect astringency expression in tea by changing the availability of free polyphenols and inhibiting polyphenol–protein binding.

Free amino acids are important contributors to the umami and sweetness of tea and are also among the key indicators in tea quality evaluation. Common free amino acids in tea include theanine, glutamic acid (Glu), aspartic acid (Asp), serine (Ser), lysine (Lys), alanine (Ala), and proline (Pro) [51,52,53]. Different amino acids contribute differently to the flavor of tea infusion. Theanine, glutamic acid, aspartic acid and other amino acids are the main compounds responsible for umami taste of tea infusion, while glycine, alanine, threonine, and other amino acids are the main compounds responsible for sweetness of tea infusion. Histidine, tryptophan, and other amino acids contribute to the bitterness of tea infusion [54,55]. Among them, L-theanine is the most abundant and is the main source of the fresh taste of tea. It is also the most widely studied representative amino acid in current research on the mechanisms of astringency regulation [56,57]. From a molecular structure perspective, amino acids contain amino (-NH_2_) and carboxyl (-COOH) groups, allowing them to form hydrogen bonds with polyphenols. Therefore, theanine may participate in polyphenol-side regulation by altering the binding state of polyphenols and can also balance bitterness in tea infusion through its umami taste.

**Figure 2:**
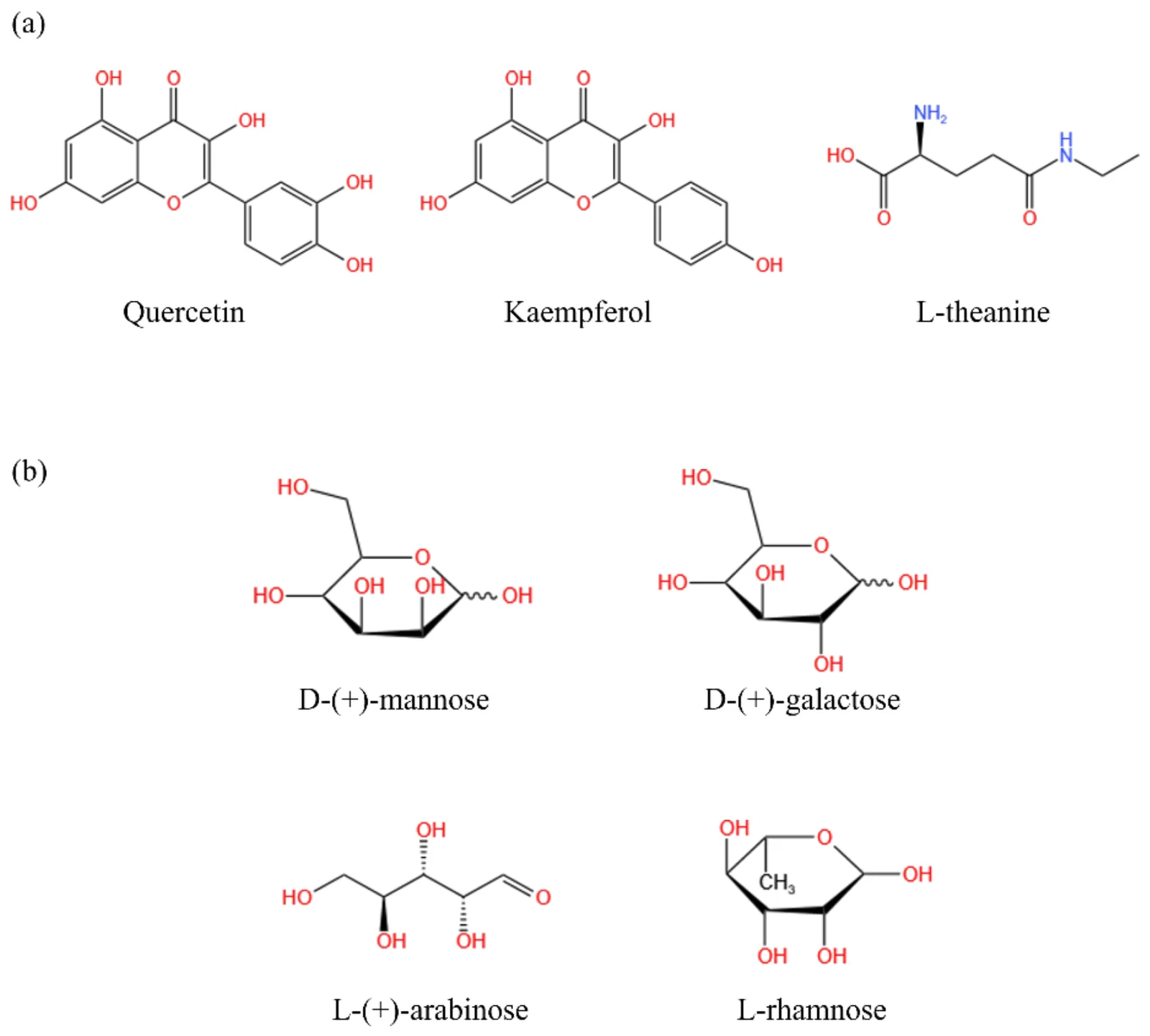
(**a**) Chemical structures of common flavonol aglycones; (**b**) Chemical structures of several common polysaccharides.

Polysaccharides are another important class of flavor-related components in tea and are widely distributed natural macromolecules [58], often occurring in the form of complex glycoproteins [59]. Based on their molecular composition and charge characteristics, polysaccharides can be classified into two major classes: neutral and acidic polysaccharides. Neutral polysaccharides are primarily composed of mannose, galactose, arabinose, and other monosaccharides and are typically electrically neutral. Acidic polysaccharides, represented by rhamnogalacturonan II (RG-II), are mostly polyelectrolytes [60,61,62]. The structures of several representative polysaccharides commonly found in tea are shown in Figure 2b. Compared with the small-molecule flavor components mentioned above, the effects of polysaccharides on astringency are more evident in terms of spatial structure, solution viscosity, and complex stability. They alleviate astringency by hindering polyphenol-protein binding or enhancing the electrostatic repulsion of complexes. Furthermore, the sensory characteristics of polysaccharides themselves can contribute to the mellow taste of tea [63,64].

Overall, other flavor components in tea are not independent of astringency but rather play an important role in shaping the overall flavor of tea infusion together with astringency. Components such as flavonol glycosides, alkaloids, amino acids, and polysaccharides differ significantly in structure, molecular weight, functional group composition, and sensory attributes. Moreover, owing to differences in cultivation environments, manufacturing processes, and other factors, the types and contents of these compounds vary among tea samples and, thus, their effects on polyphenol–protein interactions also differ. A systematic organization of these flavor components aids in understanding the formation and regulation of tea astringency from the perspective of complex flavor systems and provides an analytical foundation for summarizing how flavor compounds intervene in astringency.

**Table 1:** Common sensory chemical components in tea and their taste characteristics.

Compound Category	Name	Taste Characteristics	Analytical Techniques	Astringency Adjustment Results	Astringency Regulation Mechanism	References
Tea Polyphenols	Catechin Epicatechin Epigallocatechin Gallocatechin Catechin gallate Epicatechin gallate Epigallocatechin gallate	Dry, puckering oral sensation	Molecular monitoring, structural analysis	Increase oral friction and produce astringent taste	Rely on non-covalent binding with proteins to initiate lubrication failure, resulting in astringency	[8]
Polysaccharides	Galactose Rhamnose Arabinose	Viscosity, sweetness	Electronic tongue and sensory evaluation	Enhance oral lubricity and relieve astringency	Encapsulation mechanism or ternary mechanism, relieving astringency	[62,65,66,67,68,69]
Alkaloids	Theobromine Theophylline Caffeine	Bitterness	Molecular monitoring, electronic tongue, etc.	Concentration-dependent, relieve astringency	Occupy polyphenol active sites or block polyphenol activation receptors	[70,71,72,73]
Flavonol Glycosides	Quercetin Kaempferol	Velvety, silky astringency	spectrum technology	Combines with salivary proteins to enhance astringency	More phenolic hydroxyl groups, easier formation of compounds, enhanced lubrication failure	[74,75,76]
Amino Acids	Glutamic acid Aspartic acid Theanine	Umami	Electronic tongue, sensory evaluation, etc.	Low concentration inhibits astringency, high concentration enhances astringency	Binds with polyphenols, but concentration- dependently, for reasons unknown	[56,77]

## 4. Regulation Pathways of Astringency by Flavor Components

**Figure 3:**
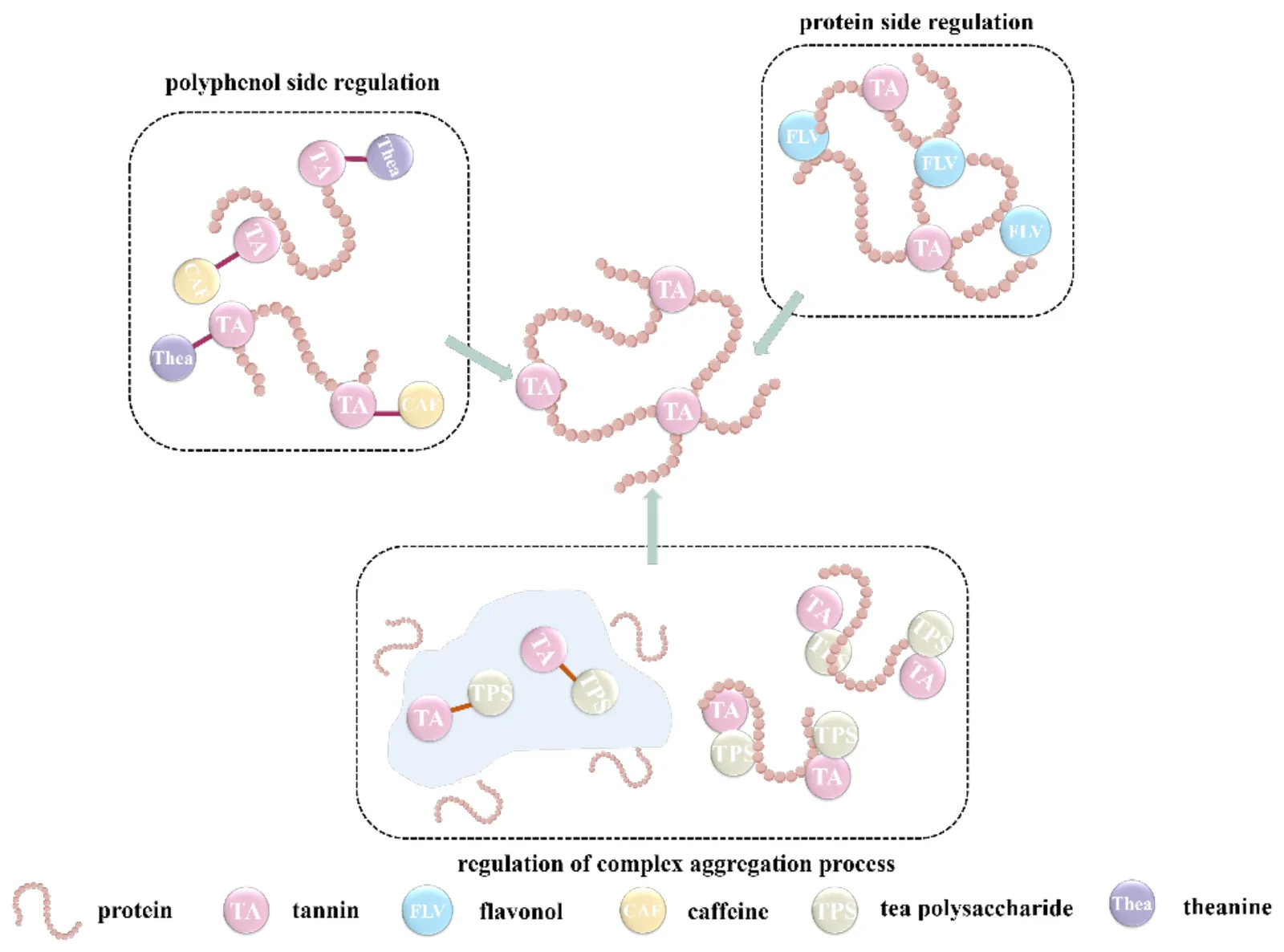
Schematic of three intervention mechanisms of flavor compounds on astringency.

The interaction between polyphenols and proteins is the core process underlying astringency generation, and other flavor components in tea regulate astringency by intervening at different stages of this process. This intervention involves complex interactions between polyphenol-protein binding and changes in the spatial structure of the complex, rather than simple enhancement or inhibition. Based on the target and mode of action, this review categorizes the regulation of astringency by other flavor components in tea into three pathways: protein-side regulation, polyphenol-side regulation, and regulation of the complex aggregation process. The schematic diagrams of the mechanisms corresponding to these three pathways are shown in Figure 3. Therefore, this chapter will focus on the three pathways mentioned above and introduce their molecular bases, representative components, and potential effects on astringency expression. It is worth noting that this chapter lists only a few representative components. For other components not mentioned here, the astringency regulation pathway should be determined based on the principal mode of molecular action and the target of the component. Molecular interaction monitoring methods such as surface plasmon resonance can be used to assist in this determination.

### 4.1. Protein-Side Regulation

In the polyphenol-protein binding process involved in the perception of astringency, salivary proteins play a crucial role [78,79]. The mechanism by which they bind to polyphenol molecules not only provides an important molecular basis for astringency formation but also creates opportunities for competition for binding sites with other flavor compounds that share similar structural characteristics. Therefore, when certain flavor compounds themselves contain structural units that can bind to proteins, they may compete with polyphenols for the limited number of protein binding sites, thereby altering the formation ratio and aggregation state of polyphenol-protein complexes and interfering with the perception of astringency [75,76].

Structurally, compounds containing phenolic hydroxyl groups and aromatic rings typically have the structural basis to interact with salivary proteins, making them more likely to act through the protein-side regulatory pathway. In the complex tea matrix, flavonol glycosides can serve as representative compounds that act through the protein-side pathway of astringency regulation. Flavonol compounds contain phenolic hydroxyl groups similar to those found in polyphenolic substances. Thermodynamic studies on flavonol–protein binding have demonstrated that this binding is largely spontaneous, driven primarily by hydrophobic interactions or hydrogen bonding [80,81]. Alcohols may compete with polyphenols for protein binding sites, increase salivary protein consumption by tea infusion, or alter the degree of complex aggregation, thereby affecting intraoral friction and modulating astringency intensity. However, the final sensory outcome of tea infusion still depends on the concentration ratio, compound structures, and complex stability [82].

The enhancement of astringency through protein-side regulation is not simply an additive increase in intensity. Compared with highly galloylated catechins, flavonols usually lack galloyl groups and contain fewer phenolic hydroxyl groups in their structures, resulting in weaker protein-binding ability and lower astringency expression intensity [83,84]. Therefore, the effects of flavonols on astringency are more likely to manifest as changes in astringency texture, softness, or duration rather than as a simple increase in astringency intensity. This also indicates that factors such as the number of hydroxyl groups in the chemical structure of the compound, the arrangement of aromatic rings, the presence of galloyl groups, and the degree of polymerization may all affect its protein binding ability, thereby altering the final regulatory effects of flavonols on astringency, including astringency intensity, duration, and perceptual characteristics.

### 4.2. Polyphenol-Side Regulation

In addition to proteins, polyphenols are important targets for other flavor compounds. The chemical structures of polyphenols contain numerous phenolic hydroxyl groups and benzene rings. The benzene rings allow π-π stacking interactions with compounds that also possess conjugated π systems, while the phenolic hydroxyls serve as hydrogen-bond donors and can form hydrogen bonds with substances containing carbonyl, amino, or hydroxyl groups [85]. Therefore, some flavor components containing the aforementioned functional groups can bind to polyphenols, and this binding may occupy the active sites that polyphenols would otherwise use for protein binding, thereby interfering with complex formation and affecting astringency expression. This pathway can be summarized as polyphenol-side regulation.

**Figure 4:**
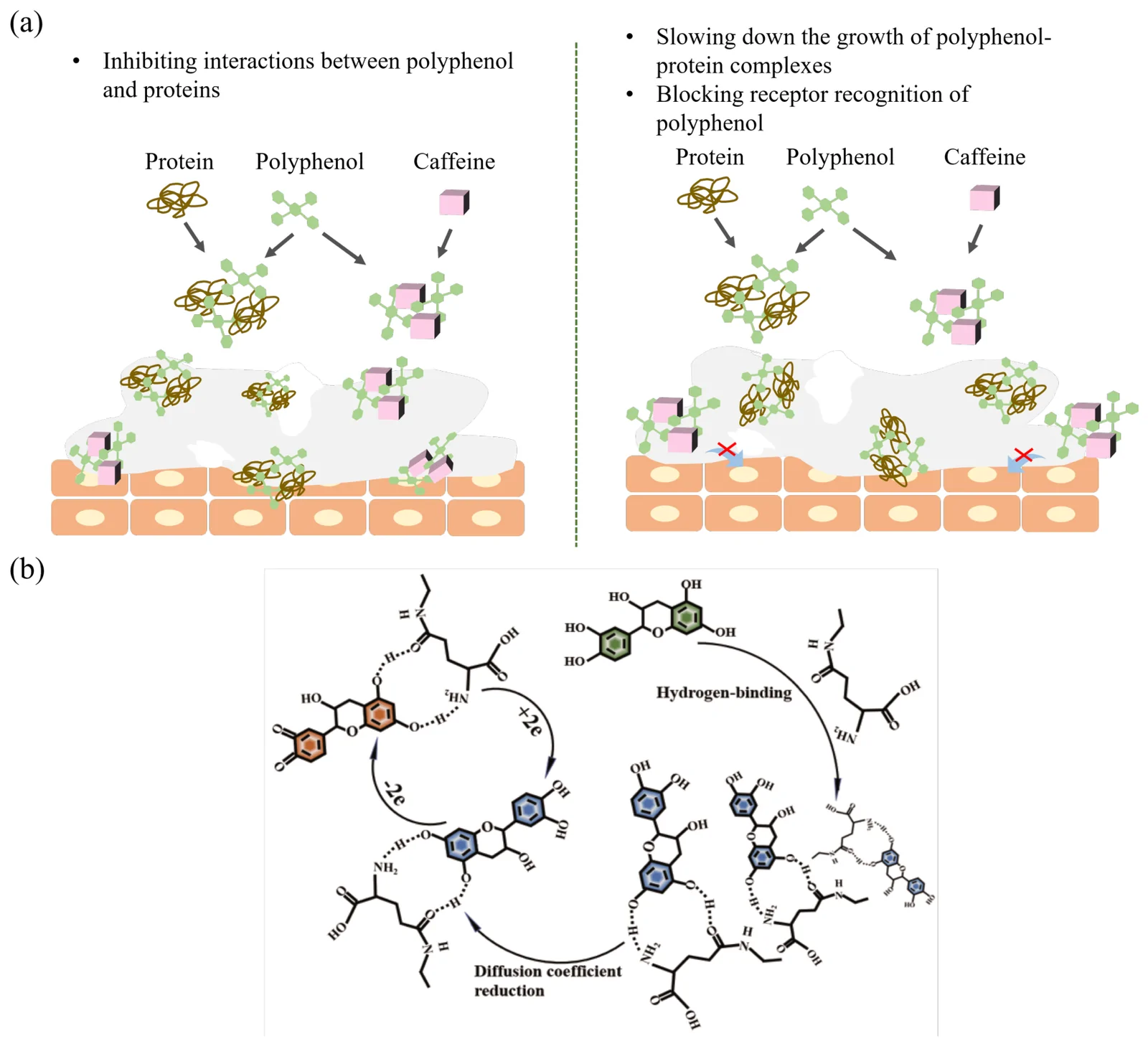
(**a**) Interference mechanism of caffeine on astringency in the salivary film network (redrawn from [72]); (**b**) Hydrogen bonding between theanine and catechins [87]. (Reproduced with permission from [87].)

As one of the most important alkaloids in tea, caffeine is a representative compound that affects astringency perception by binding to polyphenols. Current research on astringency regulation has mainly focused on the interaction between caffeine and polyphenols. Thermodynamic analysis indicates that in the binding between caffeine and polyphenols, the enthalpy change is the main driving force, suggesting that the two can bind through interactions such as hydrogen bonding, van der Waals forces, and π-π stacking [86]. Moreover, nuclear magnetic resonance (NMR) results demonstrate spatial proximity between hydrogen atoms on the methyl groups of caffeine and those on the aromatic rings of polyphenols, further confirming that they bind via π-π stacking. Therefore, caffeine in tea can compete with salivary proteins for binding sites of polyphenols, potentially altering the free state and binding ability of polyphenols. This reduces the proportion of polyphenols available to bind to salivary proteins, thereby inhibiting the astringency generation to some extent [71]. Meanwhile, sensory evaluation results also provide evidence from another perspective that the regulation of astringency by caffeine is primarily inhibitory. However, some studies have suggested that this inhibitory effect may be concentration-dependent. Specifically, it has been observed that when the concentration of polyphenols is low (less than 0.5 mg/mL), caffeine inhibits astringency by reducing the degree of lubrication failure; when the concentration of polyphenols is high (greater than 1 mg/mL), owing to the rupture of the salivary film, caffeine not only suppresses the increase in friction but also blocks the binding between polyphenols and receptors [72]. The schematic diagram of the concentration-dependent mechanism by which caffeine inhibits astringency is shown in Figure 4a. However, some studies suggest that under low-concentration astringency conditions, caffeine has almost no regulatory effect on astringency [73]. These findings indicate that the effect of caffeine on astringency is not simply inhibitory but is jointly modulated by caffeine content, polyphenol concentration, and their ratio.

In addition to caffeine, free amino acids in tea act through the polyphenol-side pathway of astringency modulation. The binding modes between amino acids and polyphenols differ depending on the type of amino acid. Hydrophobic amino acids such as leucine and proline mainly rely on entropy-driven interactions (such as hydration effects) with polyphenols, while most other amino acids primarily bind to polyphenols through enthalpy-driven interactions, namely hydrogen bonding, electrostatic attraction, and other effects [88]. Among them, theanine, the most abundant free amino acid in tea, primarily relies on its amino (-NH_2_) and carboxyl (-COOH) functional groups in its chemical structure to bond with the phenolic hydroxyl groups in polyphenols through hydrogen bonding [87], as shown in Figure 4b. Studies in specific model systems have shown that the effects of umami amino acids such as theanine on catechin-induced astringency may be concentration-dependent, and their regulatory trends are more complex. Specifically, a study that examined changes in astringency intensity under the influence of free amino acids in (-)-epigallocatechin-3-gallate(EGCG), (-)-gallocatechin-3-gallate (GCG), and (-)-epicatechin-3-gallate (ECG) systems found that in the EGCG and ECG systems, at low concentrations (less than 0.17 mg/mL), amino acids mostly exhibited an inhibitory effect on astringency. However, at medium to high concentrations (greater than 0.17 mg/mL), they exhibited an enhancing effect on astringency. The astringency regulation pattern in the GCG system is more complex. The turning point concentration of theanine is still around 0.1 mg/mL, showing inhibition at low concentrations and enhancement at high concentrations, whereas aspartic acid and glutamic acid show the opposite trend [56]. The occurrence of such changes may be related to the spatial conformation changes of polyphenol-protein complex products, or elements such as site-binding priority, but the molecular mechanism underlying its concentration dependence still requires further investigation.

Overall, flavor components involved in astringency regulation through polyphenol-side regulation often exhibit a certain concentration dependence. This may be because different pathways dominate at different concentration ranges. This phenomenon may be related to the priority of intermolecular binding, but the specific reasons remain unclear, which also points to an important direction for future research. Additionally, it is worth noting that this concentration-dependent regulatory effect may also be influenced by experimental conditions such as temperature, humidity, and pH. Furthermore, the ionic strength of the tea system, the concentrations of various components in the samples used, and the components themselves may also interfere with the results. Therefore, the applicability of the concentration-dependent sensory effects discussed in this review may be limited, and further research is needed to verify these findings.

### 4.3. Complex Aggregation Process Regulation

In addition to acting on the protein side or polyphenol side during polyphenol-protein binding, some flavor components may also affect astringency expression by influencing colloidal stability or the state of complex aggregation during the aggregation process. This pathway differs from the two previously mentioned pathways in that it primarily relies on the physical involvement of macromolecular or colloidal components in the formation of polyphenol-protein complexes. In tea, polysaccharides are representative components that regulate astringency through this pathway. Polysaccharides typically have large molecular size, strong hydration capacity, and a certain degree of viscosity. They can alleviate astringency by enhancing oral lubrication, encapsulating polyphenol molecules, and altering the aggregation state of complexes [89].

The inhibitory effect of polysaccharides on astringency is associated with their high viscosity. Astringency arises from increased friction in the oral cavity, whereas the high viscosity of polysaccharides enhances oral lubrication, reducing friction and alleviating astringency to some extent [90,91]. However, viscosity is not the only factor contributing to the alleviation of astringency by polysaccharides. The ability of polysaccharides to reduce astringency also involves factors such as molecular conformation and charge characteristics. Different polysaccharides may through different mechanisms inhibit astringency.

**Figure 5:**
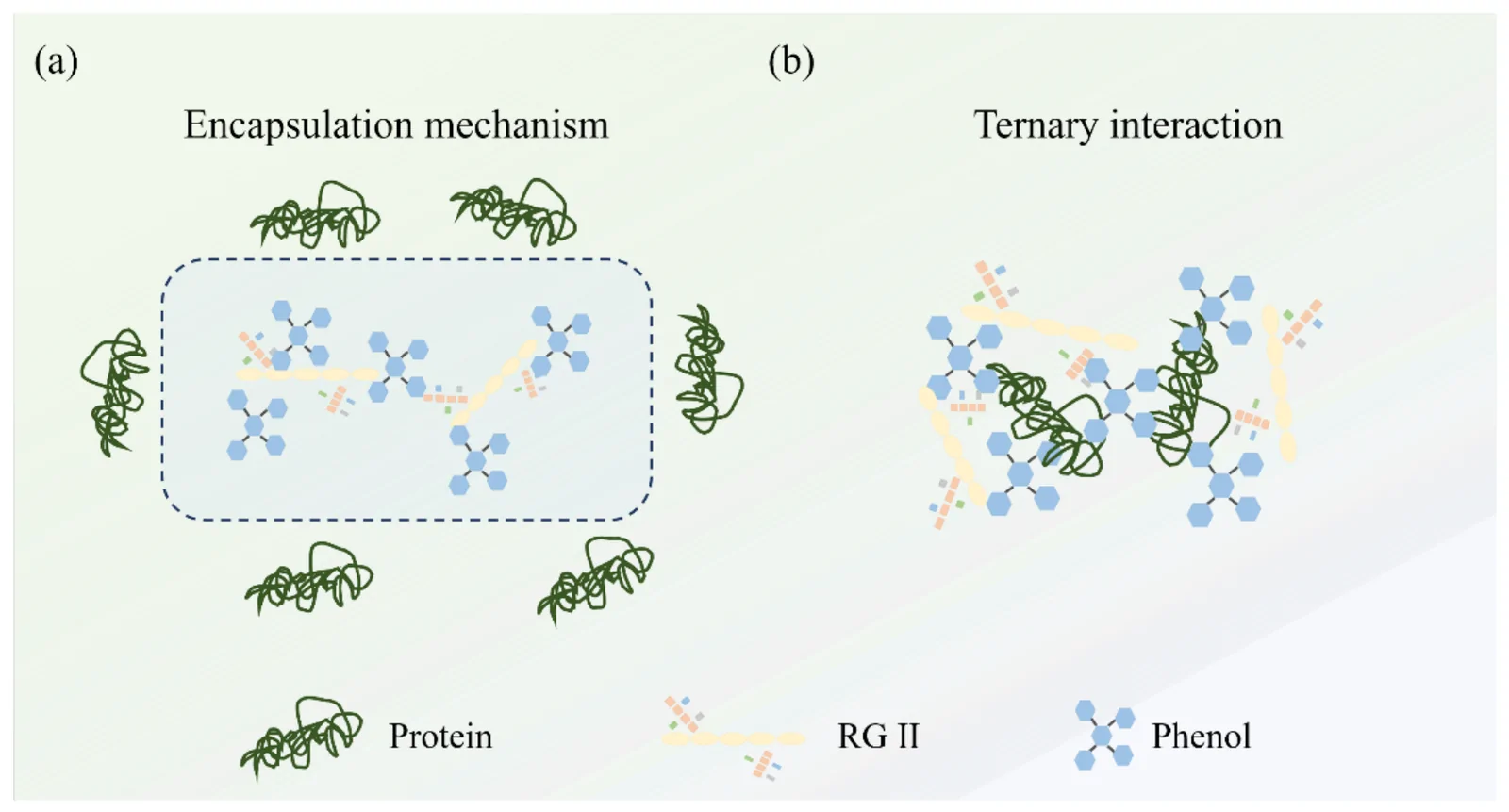
(**a**) Schematic of inclusion mechanism of polysaccharides on astringency; (**b**) Schematic of ternary mechanism of polysaccharides on astringency (Redrawn from [68]).

Regarding the mechanisms by which polysaccharides reduce astringency, existing studies typically classify them into two main types: the ternary mechanism and the inclusion mechanism (as illustrated in Figure 5a,b). The inclusion mechanism primarily stems from the spatial structures exhibited by some polysaccharides, such as helices and pores, which can encapsulate polyphenol molecules and hinder their further binding to salivary proteins, thereby alleviating astringency. Polysaccharide systems composed of monosaccharide units such as arabinose, galactose, and glucose may affect astringency expression through this mechanism [63]. The ternary mechanism involves the formation of ternary complexes among polysaccharides, polyphenols, and proteins via non-covalent interactions. Most tea infusions are acidic (pH 3–6), and RG-II carries a negative charge, so its ternary complexes with proteins and polyphenols also carry a negative charge. The electrostatic repulsion among these complexes results from their net negative charge [92,93]. Meanwhile, the Debye screening occurring within the ionic strength range typical of tea infusion is insufficient to fully eliminate this electrostatic repulsion. Therefore, it is difficult for ternary complexes to aggregate into larger precipitates, thereby reducing oral friction and producing an astringency-suppressing effect [67,68,69]. Polysaccharides capable of acting through this mechanism are mostly anionic polysaccharides, which can introduce additional negative charges onto the surface of complex particles; these are acidic polysaccharides such as RG-II and other galacturonic acid-containing polysaccharides.

In comparison, regulatory pathways involving complex aggregation processes are more evident in the regulation of complex formation and aggregation outcomes. The effect of polysaccharides on astringency should not be understood simply as an increase in viscosity; their molecular structure, charge properties, and interactions with polyphenols or proteins should also be considered. Overall, regulatory pathways involving complex aggregation processes are generally associated with astringency alleviation, but their effect intensity and manifestation are still influenced by factors such as polysaccharide type, concentration, and the oral environment.

## 5. Discussion and Perspectives

This review proposes a classification scheme for astringency regulation pathways based on interference with polyphenol–protein interactions. This classification approach helps link the structural characteristics and molecular mechanisms of action of different flavor components in tea to their impact on the sensory expression of astringency. The three pathways proposed in this review are based on their modes of action and targets. Among them, components primarily acting on salivary proteins can be categorized under protein-side regulation, components primarily acting on polyphenols can be categorized under polyphenol-side regulation, and components primarily affecting complex aggregation or altering oral lubrication effects can be categorized under complex aggregation process regulation. Therefore, in future research, sensory evaluation results, tribological information, and multiscale physicochemical information can be combined to verify the molecular interactions through which compounds such as amino acids, flavonols, and alkaloids regulate the formation of astringency, providing support for the three pathways.

These three pathways are not independent. Structurally, some compounds with phenolic hydroxyl groups can not only bind to proteins but also to polyphenols. Meanwhile, some polysaccharide components, such as guar gum and gum arabic, which are usually classified as components that regulate complex aggregation processes, may also interact with polyphenols owing to their specific spatial conformations and chemical groups, thereby exhibiting polyphenol-side regulatory characteristics. On the other hand, some compounds that primarily act on the protein side or polyphenol side may also be able to alter oral lubrication owing to their physical properties, thereby exhibiting characteristics of regulation of the complex aggregation process under specific conditions. As for the final manifestation of astringency regulation by these compounds, the key lies in which regulatory pathway predominates under the specific conditions considered. Based on this inductive framework, it is further hypothesized that at different concentrations of the same flavor component, the main stage and target of action may shift with concentration, and different dominant pathways may lead to differences in the final regulatory effect on astringency. This may also be the reason why some flavor compounds show concentration dependence.

In addition, this review still has limitations in summarizing the regulatory pathways of flavor compounds in tea on astringency. Some of the studies included in this review are heterogeneous, with differences in the choice of matrices or reference samples, the conditions used in protein model experiments, and other aspects. Moreover, the mechanistic analyses are mostly based on simplified models or other astringent systems and have not been fully extended to the tea matrix. Tea is a highly complex system, and the chemical components it contains, together with the interconversion of some components during tea processing, mean that the chemical composition of actual tea samples is not constant. Therefore, the three types of pathways proposed in the current review are mainly focused on organizing and summarizing existing evidence, and further validation is needed in complex matrices. In addition, current research mostly focuses on analyzing the mechanism of action of individual components, but in tea, these flavor components coexist, and the synergistic effects among these components, as well as their dominant relationships in regulating astringency, are still unclear. Moreover, most of the related research is limited to molecular interactions or sensory performance, with little analysis linking the two. To this end, future studies can use techniques such as surface plasmon resonance (SPR) and nuclear magnetic resonance (NMR) spectroscopy to monitor interactions involving various flavor components in tea and analyze their binding kinetics and conformational changes, or use methods such as isothermal titration calorimetry (ITC) and quartz crystal microbalance with dissipation monitoring (QCM-D) to explore changes in thermodynamic parameters, thickness, mass, and structure before and after these flavor components participate in polyphenol-protein binding. These results can be combined with information such as zeta potential, turbidity, and amount of precipitation to further explore how interactions involving these flavor components affect oral lubrication. Finally, by combining molecular interaction monitoring with sensory evaluation experiments, this approach can link mechanism-based inferences from molecular interactions to actual sensory outcomes, providing more reliable evidence for astringency regulation.

## 6. Conclusions

This review systematically outlines the regulatory mechanisms of flavor components coexisting in tea on astringency, and summarizes a conceptual framework. Overall, this review discusses the regulation of astringency by these flavor compounds from three perspectives: the protein side, the polyphenol side, and the complex aggregation process. Among them, protein-side regulation mainly involves the occupancy of salivary protein binding sites by compounds containing phenolic hydroxyl groups, such as flavonol glycosides. Polyphenol-side regulation mainly involves the binding of caffeine, amino acids, and other components to polyphenolic substances such as catechins, as well as their effects on polyphenol-protein binding. The regulation of the complex aggregation process is mainly represented by polysaccharides in tea, which affect complex aggregation by altering the spatial structure of complexes or encapsulating polyphenol molecules, thereby modulating the degree of lubrication failure. These three mechanisms do not act independently, but may jointly affect the final astringency intensity, texture, and overall flavor expression through interactions. This review summarizes the multipath regulatory mechanism of astringency, providing a reference for understanding how tea astringency forms and changes in complex flavor systems. As tea is a typical system with a well-defined chemical composition and a well-characterized astringency mechanism, the research approaches and classification framework for astringency regulation pathways in tea can also provide useful references for astringency research on other polyphenol-rich foods. Future research can move beyond single-component analysis in tea matrices by combining oral tribology, sensory evaluation, and molecular simulation to further reveal the relative dominance and interactions of various regulatory pathways when multiple components coexist, thereby laying a theoretical foundation for establishing more explanatory models of tea astringency regulation.

## Data Availability

This study did not generate any new datasets. All data analyzed are from publicly available sources, as cited in the manuscript and Supplementary Materials.

## Supplementary Materials

The following supporting information can be downloaded at: https://www.mdpi.com/article/10.3390/molecules31203659/s1.
