# Supplementary material for: Multifaceted Regulation of Tea Astringency: A Review of Intervention Pathways by Co-Existing Flavor Components
Source: Molecules. 2026 Oct 13;31(20):3659. doi: 10.3390/molecules31203659 (PMC13707796; doi:10.3390/molecules31203659)
Supplement: Supplementary file 1 [file molecules-31-03659-s001.zip › molecules-4548526-supplementary.pdf]

Supplementary Materials

Table S1 The evidence base and strength of the proposed mechanism

Verification Mechanism	experimental system	matrix	concentration range	Analytical Techniques	Level of Evidence	References
The interaction between flavonols and proteins	Bovine serum albumin (BSA), 3-hydroxyflavone (3HF)	Simplified model solution system	BSA: 0 ~ 60 μM<br>3HF: 5 μM	Optical techniques such as UV visible absorption spectroscopy and steady-state fluorescence emission spectroscopy	High	[1]
The interaction between flavonols and proteins enhances bitter taste	Human saliva, Quercetin-3-O-Glucoside	Simplified model solution system	Quercetin-3-O-Glucoside: 0-90μM	Fluorescence quenching, dynamic light scattering	High	[2]
The interaction between flavonols and proteins, and the effect of glycosylation on protein binding	Bovine serum albumin (BSA), baicalein, quercetin	Simplified model solution system	baicalein, quercetin: 1–10 μM<br>BSA:10 μM	Fluorescence spectrum, UV visible absorption spectrum	Medium	[3]
The interaction mechanism between caffeine and polyphenols	Caffeine, methyl gallate, m-nitrobenzoic acid, potassium chlorogenic acid	Solid state crystal model system	No concentration gradient experiment, all complexes are in a 1:1 stoichiometric ratio	X-ray single crystal diffraction, UV visible absorption spectroscopy	High	[4]

Table S1 (continued) The evidence base and strength of the proposed mechanism

Verification Mechanism	experimental system	matrix	concentration range	Analytical Techniques	Level of Evidence	References
Caffeine occupies some polyphenol binding sites and inhibits polyphenol protein interactions	EGCG，Caffeine, human saliva	In vitro saliva model	EGCG：0.25~ 2mg/mL Caffeine: 0.5 mg/mL	Fluorescence microscope, molecular docking, isothermal titration calorimetry, electron tongue, etc	High	[5]
The combination of umami amino acids and polyphenols affects the expression of astringency	EGCG，ECG，GCG，EGC，EC，Theanine, glutamic acid (Glu), aspartic acid (Asp)	Simplified model solution volume	Catechin monomer: 0.04-1.39 mg/mL Theanine: 3.77-14.4 mg/mL Glu, Asp: 0.26-1 mg/mL	electronic tongue	Medium	[6]
Polysaccharides alleviate astringency	Tannic acid, guar gum, xanthan gum, arabic gum, carboxymethyl cellulose	Simplified model solution volume	Tannic acid: 0.2% Guar gum: 0.14% -0.42% Xanthan gum: 0.19% -0.58% Arabic gum: 0.99% -2.98% Carboxymethyl cellulose: 1.14% -3.42%	Rotational viscometer	Medium	[7]

Table S1 (continued) The evidence base and strength of the proposed mechanism

Verification Mechanism	experimental system	matrix	concentration range	Analytical Techniques	Level of Evidence	References
Polysaccharides can inhibit astringency through a ternary mechanism	Acidic proline rich protein, P-B peptide，Proanthocyanidin dimer B2, PNG, arabinogalactan protein, rhamnose galacturonate II	Simplified model solution volume	aPRPs：6μM，15μM P-B peptide：4μM，30μM Proanthocyanins B2: 540μM, 400μM, 1000μM PNG：60μM，40μM，500μM Polysaccharides: 0-1.2 g/L	HPLC，Nephelometry，STD-NMR	upper middle	[8]
Polysaccharides inhibit astringency expression through a ternary mechanism	Alpha amylase, alkaline proline rich protein (IB8c), polysaccharides (AGP0, AGP50, AGP150, PG II)	Simplified model solution volume	Alpha amylase: 15.6 mg/L, 22.0 mg/L IB8c：31.2 mg/L，3.12 mg/L Polysaccharides: 0-0.6 g/L	90 ° light scattering	High	[9]

Literature Search Strategy

In order to ensure the comprehensiveness and objectivity of the review content, the authors systematically searched the Web of Science and Google Scholar databases. Since some literature related to sensory mechanisms might have been published relatively earlier, when conducting the literature search, the time range for paper publication was not specified to obtain more comprehensive and accurate authoritative information on the mechanisms. The main search terms used during the search were: tea astringency, astringency perception, flavor components in tea, flavonol glycosides and protein, flavonol glycosides and astringency, flavonol glycosides in tea, alkaloid and astringency, alkaloid in tea, alkaloid and polyphenol, amino acid and astringency, amino acid in tea, amino acid and polyphenol, polysaccharide and astringency, polysaccharide in tea, etc. The latest search date of the article is 2026.07.23.

When writing the review, appropriate articles were selected based on the research needs and the requirement that the combination process of polyphenols and proteins or a certain component in the combination, the component selected in the research needs to be able to affect or intervene in the perception of astringency, the components studied in the article are related to the components contained in tea, and so on. Original research and reviews published in peer-reviewed journals were selected. Some conferences, letters, and dissertations were excluded. The references cited are all English papers. In the selection of papers, considering that the review selects to explain the regulation of astringency by other flavors from the perspective of mechanism, when selecting references for the regulation of astringency path by flavor components, it will not be limited to the tea system. Papers that meet the selected criteria in any system will be included in the selection.

During the literature screening process, given the limited number of relevant studies, some evidence related to mechanisms or component descriptions would not be confined to the tea system, but rather focused on demonstrating the interactions between molecules. Ultimately, this paper initially retrieved 166 articles, and after screening, 84 references were selected for inclusion.

1. Guharay, J.; Sengupta, B.; Sengupta, P., Protein–flavonol interaction: fluorescence spectroscopic study. *Proteins: Structure, Function, and Bioinformatics* **2001,** 43, (2), 75-81.
2. Ferrer-Gallego, R.; Brás, N. F.; García-Estévez, I.; Mateus, N.; Rivas-Gonzalo, J. C.; de Freitas, V.; Escribano-Bailón, M. T., Effect of flavonols on wine astringency and their interaction with human saliva. *Food chemistry* **2016,** 209, 358-364.
3. Cao, H.; Wu, D.; Wang, H.; Xu, M., Effect of the glycosylation of flavonoids on interaction with protein. *Spectrochimica Acta Part A: Molecular and Biomolecular Spectroscopy* **2009,** 73, (5), 972-975.
4. Charlton, A. J.; Baxter, N. J.; Khan, M. L.; Moir, A. J.; Haslam, E.; Davies, A. P.; Williamson, M. P., Polyphenol/peptide binding and precipitation. *Journal of agricultural and food chemistry* **2002,** 50, (6), 1593-1601.
5. Zhou, Z.; Ou, M.; Shen, W.; Jin, W.; Yang, G.; Huang, W.; Guo, C., Caffeine weakens the astringency of epigallocatechin gallate by inhibiting its interaction with salivary proteins. *Food Chemistry* **2024,** 460, 140753.
6. Liu, Z.; Ran, Q.; Li, Q.; Yang, T.; Dai, Y.; Zhang, T.; Fang, S.; Pan, K.; Long, L., Interaction between major catechins and umami amino acids in green tea based on electronic tongue technology. *Journal of Food Science* **2023,** 88, (6), 2339-2352.
7. Troszyńska, A.; Narolewska, O.; Robredo, S.; Estrella, I.; Hernández, T.; Lamparski, G.; Amarowicz, R., The effect of polysaccharides on the astringency induced by phenolic compounds. *Food Quality and Preference* **2010,** 21, (5), 463-469.
8. Brandão, E.; Silva, M. S.; García-Estévez, I.; Williams, P.; Mateus, N.; Doco, T.; de Freitas, V.; Soares, S., The role of wine polysaccharides on salivary protein-tannin interaction: A molecular approach. *Carbohydrate polymers* **2017,** 177, 77-85.
9. Carvalho, E.; Mateus, N.; Plet, B.; Pianet, I.; Dufourc, E.; De Freitas, V., Influence of wine pectic polysaccharides on the interactions between condensed tannins and salivary proteins. *Journal of Agricultural and Food Chemistry* **2006,** 54, (23), 8936-8944.
